# Dominant Follicle Size as a Predictor of Pregnancy in Letrozole Intrauterine Insemination

**DOI:** 10.3390/jcm15155857

**Published:** 2026-07-27

**Authors:** Emel Özalp, Belgin Savran Üçok, Türkan Dikici Aktaş, Recep Taha Ağaoğlu, Özgür Volkan Akbulut, Kubilay Çanga, İnci Kahyaoğlu

**Affiliations:** Department of Obstetrics and Gynecology, Ministry of Health, Ankara Etlik City Hospital, 06170 Ankara, Turkey; drbelgin@gmail.com (B.S.Ü.); trkndkc1@gmail.com (T.D.A.); tahaagaoglu@hotmail.com (R.T.A.); akbulutvolkan@yahoo.com (Ö.V.A.); kblycng@gmail.com (K.Ç.); mdincikahyaoglu@gmail.com (İ.K.)

**Keywords:** aromatase inhibitor, ovarian stimulation, polycystic ovary syndrome, folliculometry, unexplained infertility, subfertility

## Abstract

**Background/Objectives:** Letrozole combined with intrauterine insemination (IUI) is a common first-line treatment for unexplained and anovulatory infertility; however, the optimal dominant follicle size at human chorionic gonadotrophin (hCG) triggering remains uncertain. We evaluated associations of follicle size with biochemical pregnancy, clinical pregnancy, and live birth. **Methods:** This retrospective cohort included 596 letrozole–IUI cycles from 454 women aged <40 years treated between May 2024 and August 2025. Cycles were grouped by dominant follicle diameter at trigger (17.0–18.9, 19.0–21.0, and >21.0 mm). Mixed-effects logistic regression accounted for repeated cycles. **Results:** Biochemical pregnancy, clinical pregnancy, and live-birth rates differed across groups and were highest at 19.0–21.0 mm; live-birth rates were 3.4%, 15.1%, and 8.6%, respectively (*p* = 0.008). Compared with 17.0–18.9 mm, the 19.0–21.0 mm group had higher adjusted odds of clinical pregnancy (aOR 5.58, 95% CI 1.31–23.84; *p* = 0.020) and live birth (aOR 15.15, 95% CI 1.74–131.95; *p* = 0.014), although both estimates were imprecise. Adjusted live-birth probabilities were 3.5%, 16.1%, and 9.1%, respectively. The direction of the live-birth association was preserved in the single-cycle sensitivity analysis (aOR 3.85, 95% CI 1.22–12.13; *p* = 0.021). Continuous linear and quadratic follicle-diameter models were not significant. **Conclusions:** The 19.0–21.0 mm category was associated with higher reproductive outcome rates, including live birth; however, the observational design, sparse events, wide confidence intervals, and null continuous analyses preclude defining an optimal trigger threshold. These findings are hypothesis-generating and require prospective multicenter confirmation.

## 1. Introduction

Infertility is defined as the failure to achieve a pregnancy after 12 months of regular unprotected intercourse [1]. It affects approximately 15–20% of couples of reproductive age and remains a major public health issue [2]. Infertility has a multifactorial etiology, with male factor (~35%), tubal pathology (~15%), and ovulatory dysfunction (~25%) among the leading causes. Despite comprehensive evaluation, no definitive cause is identified in nearly 30% of cases, which are classified as unexplained infertility. Anovulatory infertility, often associated with polycystic ovary syndrome (PCOS), is also among the most common causes of female infertility and accounts for approximately 25–40% of cases [2,3,4,5].

In clinical practice, infertility management is typically approached in a stepwise manner, progressing from less invasive treatments to assisted reproductive technologies, depending on patient age and underlying etiology. One of the most frequently used first-line strategies is ovulation induction (OI) combined with intrauterine insemination (IUI), particularly for couples with ovulatory dysfunction or unexplained infertility [5]. Clomiphene citrate (CC), a selective estrogen receptor modulator, has long been used for OI by blocking hypothalamic estrogen receptors and increasing gonadotropin secretion; however, its anti-estrogenic effects may adversely affect endometrial thickness and cervical mucus.

Letrozole, a third-generation aromatase inhibitor initially developed for the adjuvant treatment of postmenopausal breast cancer, has become a strong alternative to CC in infertility practice. By inhibiting the conversion of androgens to estrogens, letrozole reduces negative feedback on the hypothalamic–pituitary axis and increases endogenous gonadotropin secretion [6]. Unlike CC, it does not deplete estrogen receptors, is generally associated with better preservation of endometrial receptivity, and tends to support monofollicular development. Randomized controlled trials and evidence-based guidelines support letrozole as an effective first-line ovulation induction agent in anovulatory women with PCOS, with higher live-birth rates compared with CC [3,7,8,9].

Pregnancy outcomes in OI–IUI cycles are influenced by a heterogeneous set of factors, including female and male age, body mass index (BMI), baseline hormonal parameters, infertility duration, the pharmacologic agent used for OI, endometrial thickness, post-preparation total motile sperm count (TMSC), and the characteristics of the dominant follicle at the time of trigger [10,11,12,13,14]. Follicle size is an indirect marker of oocyte maturity; the timing of human chorionic gonadotropin (hCG) trigger, based on follicle measurements, is considered a critical component of cycle management. Nevertheless, there remains no consensus regarding the optimal dominant follicle size at the time of hCG trigger in letrozole-related IUI cycles. Reported optimal sizes vary widely across studies, ranging from 16.1–18.0 mm in letrozole–human menopausal gonadotrophin (HMG) IUI cycles [15] to 19.1–21.0 mm in first-cycle letrozole–IUI [13], 19–23 mm in mono-follicular letrozole–IUI [16], and 23–28 mm in CC or letrozole IUI cycles [17]. Moreover, emerging evidence suggests that the optimal trigger follicle size may differ by infertility etiology, with differential patterns reported between ovulatory dysfunction and unexplained infertility in letrozole–IUI cycles [18].

Biologically, follicular diameter reflects coordinated follicular-fluid accumulation, granulosa-cell steroidogenesis, cumulus expansion, and oocyte nuclear and cytoplasmic maturation. Triggering too early may precede the acquisition of full oocyte competence, whereas excessive delay may increase the risk of post maturity or atresia, and may impair synchronization between ovulation and endometrial receptivity. In letrozole-stimulated cycles, the relatively short half-life and preservation of estrogen receptors may support endometrial development; however, the lower trigger-day estradiol concentrations typical of aromatase inhibition may alter the relationship between measured follicle size and biological maturity. A mid-range diameter could, therefore, represent a clinically plausible balance between oocyte competence and endometrial timing, although this hypothesis requires direct testing [13,15,16,17,18].

Given these inconsistencies and the heterogeneity of stimulation regimens, patient populations, and outcome definitions across prior studies, additional data from well-defined cohorts using standardized protocols are needed. This retrospective cohort study, therefore, evaluated the associations of dominant follicle size on the day of hCG trigger with biochemical pregnancy, clinical pregnancy, and live birth in women with unexplained or anovulatory infertility undergoing letrozole-induced OI followed by IUI. We also assessed whether the category-based findings were supported by continuous linear and quadratic models and by sensitivity analyses addressing repeated treatment cycles.

## 2. Materials and Methods

### 2.1. Study Design and Setting

This retrospective cohort study was conducted at the Assisted Reproductive Technologies Unit of Ankara Etlik City Hospital (Ankara, Turkiye), a tertiary referral center. Cycles performed between May 2024 and August 2025 were evaluated. Ethical approval was obtained from the institutional ethics committee (approval number: AEŞH-BADEK2-2025-498 date: 30 September 2025). All procedures were carried out in accordance with the Declaration of Helsinki. Because of the retrospective design and the use of anonymized data, the requirement for written informed consent was waived.

### 2.2. Study Population and Eligibility Criteria

The unit of analysis was the treatment cycle. Cycles were eligible for inclusion if all of the following criteria were met: (i) female age < 40 years; (ii) ovulation induction with letrozole followed by intrauterine insemination (IUI) for either ovulatory dysfunction (anovulatory infertility) or unexplained infertility; (iii) at least one patent fallopian tube documented by hysterosalpingography or laparoscopy; and (iv) semen parameters within World Health Organization reference limits, with a post-preparation total motile sperm count (TMSC) of 10 million or higher.

Cycles were excluded if basal follicle-stimulating hormone (FSH) was >10 mIU/mL, if a multifollicular response occurred (more than two follicles measuring 14 mm or larger), or if the patient had a history of ovarian or tubal surgery, endometriosis, adenomyosis, intrauterine adhesions, congenital uterine or cervical anomalies, fetal anomaly or recurrent pregnancy loss, or previous or current malignancy. Each participant was assigned a unique study identifier to preserve confidentiality during data handling and analysis.

### 2.3. Ovarian Stimulation, Monitoring, and Ovulation Triggering

Baseline transvaginal ultrasonography (TVUS) was performed on cycle day 2 or 3 using a Voluson S10 ultrasound system (GE HealthCare, Zipf, Austria) to confirm the absence of an ovarian cyst larger than 10 mm and to document a baseline endometrial thickness < 5 mm. Oral letrozole was administered at 5 mg/day (2.5 mg twice daily; Femara, Novartis Pharma AG, Basel, Switzerland) for five consecutive days (cycle days 3–7). Follicular monitoring was initiated on cycle day 10 and continued with serial TVUS examinations until follicular maturation.

Ovulation was triggered when at least one dominant follicle reached a diameter of 17 mm or larger, using 250 microgram recombinant human chorionic gonadotropin (hCG; choriogonadotropin alfa; Ovitrelle, Merck Europe B.V., Amsterdam, the Netherlands) administered subcutaneously.

### 2.4. Insemination Procedure, Sperm Preparation, and Luteal Support

A single IUI was performed approximately 36 h after hCG administration. A prepared sperm suspension was introduced into the uterine cavity using a sterile insemination catheter, with the catheter tip positioned near the uterine fundus, according to routine practice.

Semen samples were processed in the andrology laboratory using either density-gradient centrifugation or a swim-up technique, in accordance with standard laboratory procedures. Luteal phase support was provided in all cycles with 200 mg vaginal micronized progesterone once daily(Progestan, Koçak Farma İlaç ve Kimya Sanayi A.Ş., Istanbul, Türkiye), starting on the day of IUI.

### 2.5. Follicle-Size Grouping

Cycles were stratified according to the dominant follicle diameter on the day of hCG trigger into three predefined groups:

- Group 1: 17.0–18.9 mm;
- Group 2: 19.0–21.0 mm;
- Group 3: >21.0 mm.

The categories were prespecified before the outcome analyses and were not selected through data-driven cut-point optimization. The 17.0–18.9 mm category represented triggering shortly after the protocol’s minimum threshold of 17 mm; the 19.0–21.0 mm category represented an intermediate follicular-maturity range commonly examined in letrozole–IUI studies; and the >21.0 mm category represented triggering at a larger dominant follicle diameter. These clinically interpretable ranges were retained as the primary exposure categories, while continuous linear and quadratic models were used as sensitivity analyses [13,15,16,17,18].

### 2.6. Data Collection and Study Variables

Demographic variables included female age (years), body mass index (BMI; kg/m^2^), and duration of infertility (years). Hormonal and laboratory parameters obtained on cycle days 3–5 included FSH, luteinizing hormone (LH), prolactin, thyroid-stimulating hormone (TSH), anti-Mullerian hormone (AMH), and estradiol.

Cycle- and IUI-related variables included duration of stimulation, dominant follicle diameter on the trigger day, the presence and diameter of a secondary follicle, endometrial thickness, and post-preparation total motile sperm count (TMSC; ×10^6^). Double ovulation was defined as the presence of a secondary follicle measuring 14 mm or larger on the day of hCG trigger; cycles with a single dominant follicle or a secondary follicle < 14 mm were classified as mono-ovulatory.

### 2.7. Outcome Definitions

Biochemical pregnancy was defined as a serum beta-hCG concentration of 25 mIU/mL or higher measured 14 days after IUI. Clinical pregnancy was defined as ultrasonographic visualization of an intrauterine gestational sac on TVUS. Live birth was defined as the delivery of at least one live-born infant.

### 2.8. Study Outcomes

The prespecified primary outcome was biochemical pregnancy. Clinical pregnancy and live birth were evaluated as secondary outcomes. Because complete pregnancy follow-up was available, live birth was included as the key clinically relevant secondary outcome rather than adding a separate, retrospectively defined ongoing-pregnancy endpoint. Additional objectives were to identify factors associated with each reproductive outcome and to evaluate their relationships with dominant follicle-size categories.

### 2.9. Sample Size Estimation

This retrospective study was intended as a census of all consecutive eligible cycles during the prespecified study period; no sampling scheme or prospectively determined recruitment target was applied. Because no previous study used identical eligibility criteria and follicle-size categories, a conservative small effect (w = 0.15) was used to assess the statistical adequacy of the available cohort. Assuming a two-sided type I error rate of 0.05, 90% power, and two degrees of freedom, G*Power version 3.1.9.7 (Heinrich Heine University Düsseldorf, Düsseldorf, Germany) yielded a reference minimum of 563 cycles. This calculation was used only to contextualize the available sample and did not determine enrollment; all 596 eligible cycles were included.

### 2.10. Statistical Analysis

Statistical analyses were performed using Stata/SE, version 19.0 (StataCorp LLC, College Station, TX, USA).Continuous variables are presented as mean ± standard deviation or median (25th–75th percentile), as appropriate, and categorical variables as numbers (percentage). Distributional characteristics of continuous variables were assessed using histograms and Q–Q plots. The variables required for the primary and sensitivity analyses were complete (0% missingness); therefore, all analyses used complete-case data and no imputation was required or performed. To account for repeated IUI cycles contributed by the same participant, mixed-effects models with a patient-level random intercept were used.

Between-group comparisons of continuous variables were performed using linear mixed-effects regression models; logarithmic transformation was applied when required, based on distributional assessment. Categorical outcomes were compared using mixed-effects logistic regression models. Overall differences across follicle-size categories were evaluated using Wald χ^2^ tests; prespecified odds-ratio contrasts were derived from linear combinations of model coefficients. For the three pairwise comparisons of adjusted marginal probabilities within each outcome, *p* values were adjusted using the Bonferroni method.

Associations of candidate variables with biochemical pregnancy, clinical pregnancy, and live birth were initially examined using univariable mixed-effects logistic regression. Variables with *p* < 0.20 in univariable analyses and/or established clinical relevance were considered for inclusion in the multivariable models. The final models were kept parsimonious; automated stepwise variable-selection procedures were not used. The biochemical-pregnancy model included 129 events and seven non-intercept fixed-effect coefficients, corresponding to 18.4 events per coefficient. The clinical-pregnancy model included 78 events and six fixed-effect coefficients, corresponding to 13.0 events per coefficient, whereas the live-birth model included 74 events and six fixed-effect coefficients, corresponding to 12.3 events per coefficient.

Mixed-effects logistic regression models were estimated using adaptive Gauss–Hermite quadrature. Numerical stability was assessed by repeating the analyses using 15 and 30 integration points and comparing regression coefficients, standard errors, random-intercept variances, and log-likelihood values. All final models were estimated using 30 integration points. Population-averaged generalized estimating equation models with a binomial distribution, logit link, exchangeable working-correlation structure, and robust standard errors were additionally fitted as sensitivity analyses.

Because the categorization of a continuous predictor may result in information loss and obscure nonlinear associations, dominant follicle diameter was additionally modeled as a continuous variable in otherwise identical adjusted mixed-effects logistic regression models. Potential nonlinearity was evaluated by adding a quadratic follicle-diameter term; the corresponding linear and quadratic models were compared using likelihood-ratio tests. The results of these analyses are presented in Supplementary Table S4.

Potential effect modification by infertility etiology was explored for live birth by including an interaction term between infertility etiology and follicle-size category. Although 60 cycles from women with unexplained infertility were present in the 17.0–18.9 mm category, no live births occurred in this cell (0/60), producing complete separation and preventing reliable estimation of the full three-category interaction model. The exploratory interaction analysis was, therefore, restricted to the 19.0–21.0 mm and >21.0 mm categories, which were represented in both infertility-etiology groups.

Model diagnostics were performed separately for each final multivariable model. Multicollinearity among the fixed-effect predictors was assessed using variance inflation factors derived from auxiliary regression models containing the same fixed-effect terms. Linearity of continuous predictors on the logit scale was evaluated by adding centered quadratic terms individually to the corresponding models. Because TMSC was right-skewed and its quadratic term approached statistical significance, an additional sensitivity analysis using log-transformed TMSC was performed; alternative specifications were compared using the Akaike and Bayesian information criteria.

Potentially influential observations were evaluated using conditional Pearson and deviance residuals, with absolute values > 3 considered potentially unusual. Standardized empirical Bayes estimates of the patient-level random intercepts were also examined. Clusters with absolute standardized estimates > 3 were assessed in leave-one-cluster-out sensitivity analyses. Model calibration was evaluated using Brier scores and by comparing observed with population-averaged marginally predicted outcome probabilities, both overall and across quintiles of predicted risk. Convergence was confirmed from the optimization output and the absence of convergence or non-concavity warnings.

Adjusted marginal probabilities of biochemical pregnancy, clinical pregnancy, and live birth were calculated for each follicle-size category from the corresponding multivariable mixed-effects models. Absolute probability differences between categories were estimated using linear contrasts. Sensitivity analyses restricted to participants contributing a single IUI cycle were performed using single-level logistic regression models; Firth’s penalized likelihood method was used for clinical pregnancy and live birth to reduce potential small-sample bias. All tests were two-sided and *p* < 0.05 was considered statistically significant, unless otherwise specified.

## 3. Results

**Figure 1:**
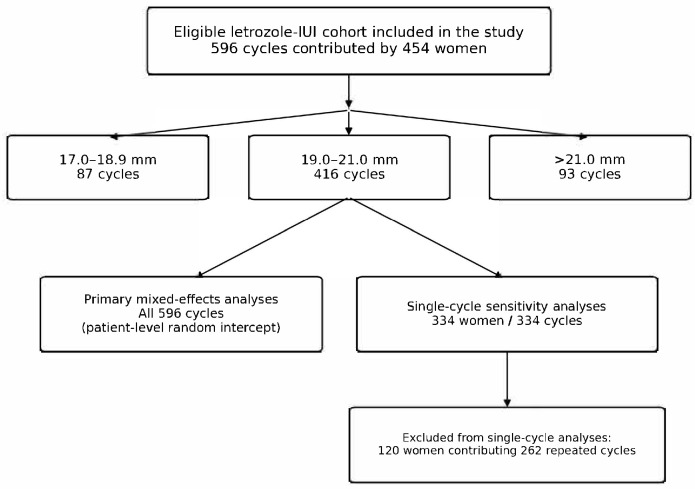
Study cohort and analytical flow diagram.

A total of 596 cycles contributed by 454 women were included. The mean number of cycles per woman was 1.31 ± 0.56, with a median of one cycle (interquartile range, 1–2). Overall, 334 women (73.6%) contributed one cycle, 99 (21.8%) contributed two cycles, 20 (4.4%) contributed three cycles, and one (0.2%) contributed four cycles. Thus, 120 women (26.4%) contributed more than one cycle and the maximum contribution from one participant was four cycles. The cohort and analytical pathways are summarized in Figure 1.

The 596 cycles were categorized according to dominant follicle size as 17.0–18.9 mm (*n* = 87), 19.0–21.0 mm (*n* = 416), and >21.0 mm (*n* = 93). Baseline demographic, hormonal, and cycle-related characteristics are presented in Table 1. No statistically significant between-group differences were observed in age, body mass index, infertility duration, basal estradiol, follicle-stimulating hormone, luteinizing hormone, prolactin, thyroid-stimulating hormone, endometrial thickness, ovulation-induction duration, or total motile sperm count (TMSC) (all *p* > 0.05). Serum anti-Müllerian hormone differed across groups (Wald χ^2^(2) = 7.69; *p* = 0.021), whereas infertility etiology and subdominant-follicle prevalence were comparable. Biochemical pregnancy, clinical pregnancy, and live-birth rates differed significantly across groups (*p* = 0.015, *p* = 0.012, and *p* = 0.008, respectively), with the highest rates in the 19.0–21.0 mm group. Live-birth rates were 3.4%, 15.1%, and 8.6%, respectively.

Univariable and multivariable mixed-effects logistic regression analyses for predictors of biochemical pregnancy are shown in Table 2. In univariable analyses, infertility type, follicular diameter, and age were associated with biochemical pregnancy (*p* < 0.05). In the multivariable mixed-effects model, infertility type (anovulatory vs. unexplained) remained independently associated with biochemical pregnancy (adjusted odds ratio [aOR] 1.794, 95% CI 1.080–2.978; *p* = 0.024). Compared with cycles with a dominant follicle size of 17.0–18.9 mm, cycles with a dominant follicle size of 19.0–21.0 mm showed higher odds of biochemical pregnancy (aOR 2.433, 95% CI 1.163–5.090; *p* = 0.018). The comparison between 17.0–18.9 mm and >21.0 mm yielded a borderline association (aOR 0.520, 95% CI 0.269–1.007; *p* = 0.052). Other covariates were not independently associated with biochemical pregnancy in the multivariable model.

Univariable and multivariable mixed-effects logistic regression analyses for predictors of clinical pregnancy are presented in Table 3. In univariable analyses, infertility type, follicular diameter, and age were associated with clinical pregnancy (*p* < 0.05). In the multivariable mixed-effects model, infertility type (anovulatory vs. unexplained) remained independently associated with clinical pregnancy (aOR 2.724, 95% CI 1.165–6.368; *p* = 0.021). Compared with cycles with a dominant follicle size of 17.0–18.9 mm, cycles with a dominant follicle size of 19.0–21.0 mm demonstrated higher odds of clinical pregnancy (aOR 5.584, 95% CI 1.308–23.843; *p* = 0.020). The comparison between 19.0–21.0 mm and >21.0 mm did not reach statistical significance in the multivariable model (*p* = 0.095). Age showed a borderline association with clinical pregnancy after adjustment.

No problematic multicollinearity was identified in the final models; mean VIF values ranged from 1.33 to 1.41, and the maximum VIF was 1.77. Centered quadratic terms provided no evidence of significant departure from linearity for the continuous predictors included in the clinical-pregnancy and live-birth models. In the biochemical-pregnancy model, the quadratic terms for endometrial thickness, AMH, and maternal age were not significant, whereas the TMSC term was borderline (*p* = 0.054). A sensitivity analysis using log-transformed TMSC modestly improved model fit but did not materially alter the follicle-size estimates. Continuous linear and quadratic analyses of dominant follicle diameter are presented in Supplementary Table S4.

No observations had absolute conditional Pearson or deviance residuals greater than three. One patient contributing four cycles had a standardized empirical Bayes random-intercept estimate greater than three in the clinical-pregnancy and live-birth models. Exclusion of this patient did not materially alter the principal estimates; the adjusted odds ratios for the 19–21 mm group remained significant for both clinical pregnancy (aOR 4.96; *p* = 0.027) and live birth (aOR 13.90; *p* = 0.019).

Agreement between observed and marginally predicted outcome probabilities was generally satisfactory. The observed versus predicted rates were 21.6% versus 21.8% for biochemical pregnancy, 13.1% versus 13.6% for clinical pregnancy, and 12.4% versus 13.2% for live birth. The corresponding Brier scores were 0.1626, 0.1098, and 0.1046. Across predicted-risk quintiles, the maximum absolute observed–predicted differences were 4.0, 2.3, and 4.0 percentage points, respectively, with no evidence of major systematic miscalibration. All models converged successfully; parameter estimates were unchanged or differed negligibly when the number of quadrature points was increased from 15 to 30.

Univariable and multivariable mixed-effects logistic regression analyses of factors associated with live birth are presented in Table 4. In the univariable analyses, follicular diameter groups were significantly associated with live birth (overall *p* = 0.047). Compared with the reference group (17–18.9 mm), the 19–21 mm group had significantly higher odds of live birth (OR 14.10, 95% CI 1.51–131.89; *p* = 0.020), whereas the >21.0 mm group did not differ significantly from the reference group (OR 4.58, 95% CI 0.47–44.55; *p* = 0.190). No significant difference was observed between the >21.0 mm and 19–21 mm groups (OR 0.32, 95% CI 0.07–1.41; *p* = 0.133). The wide confidence intervals indicated substantial uncertainty regarding the magnitude of these associations. In a sensitivity analysis using population-averaged GEE with robust standard errors, the direction of the findings remained consistent (overall *p* = 0.007). The 19–21 mm group had significantly higher odds of live birth than the reference group (OR 4.59, 95% CI 1.59–13.31; *p* = 0.005), while no significant differences were found between the >21.0 mm group and either the reference group (OR 2.45, 95% CI 0.72–8.34; *p* = 0.151) or the 19–21 mm group (OR 0.53, 95% CI 0.26–1.10; *p* = 0.090).

In the multivariable mixed-effects logistic regression analysis adjusted for infertility type, ovulation induction duration, maternal age, and AMH level, follicular diameter groups were significantly associated with live birth (overall *p* = 0.033). Compared with the 17–18.9 mm reference group, the 19–21 mm group had significantly higher odds of live birth (aOR 15.15, 95% CI 1.74–131.95; *p* = 0.014), whereas the >21.0 mm group did not differ significantly from the reference group (aOR 4.99, 95% CI 0.55–45.42; *p* = 0.154). There was also no significant difference between the >21.0 mm and 19–21 mm groups (aOR 0.33, 95% CI 0.08–1.33; *p* = 0.118). The wide confidence intervals indicated substantial uncertainty regarding the magnitude of the mixed-effects estimates. In the sensitivity analysis using population-averaged GEE with robust standard errors, the overall association remained statistically significant (overall *p* = 0.006). The 19–21 mm group had significantly higher adjusted odds of live birth than the 17–18.9 mm group (aOR 4.95, 95% CI 1.69–14.48; *p* = 0.003), while no significant differences were observed between the >21.0 mm and 17–18.9 mm groups (aOR 2.65, 95% CI 0.78–9.09; *p* = 0.120) or between the >21.0 mm and 19–21 mm groups (aOR 0.54, 95% CI 0.26–1.11; *p* = 0.095).

There were 60 cycles from women with unexplained infertility in the 17.0–18.9 mm category; however, no live births occurred in this subgroup (0/60), resulting in complete separation and preventing reliable estimation of the corresponding interaction parameter. Therefore, a complete interaction analysis across all three follicle-size categories could not be estimated. In the exploratory analysis restricted to the 19.0–21.0 mm and >21.0 mm categories, there was no statistically significant interaction between follicle-size category and infertility etiology for live birth (interaction OR 0.75, 95% CI 0.05–10.72; *p* for interaction = 0.834).

In sensitivity analyses treating dominant follicle diameter as a continuous predictor, no significant linear association was observed with biochemical pregnancy (adjusted OR per 1-mm increase 1.01, 95% CI 0.88–1.16; *p* = 0.858), clinical pregnancy (adjusted OR 1.01, 95% CI 0.81–1.25; *p* = 0.933), or live birth (adjusted OR 1.07, 95% CI 0.83–1.38; *p* = 0.601). The quadratic follicle-diameter term was also not statistically significant for biochemical pregnancy (*p* = 0.279), clinical pregnancy (*p* = 0.476), or live birth (*p* = 0.195). Quadratic models did not significantly improve model fit compared with the corresponding linear models (likelihood-ratio *p* = 0.262, *p* = 0.464, and *p* = 0.158, respectively) (Supplementary Table S4).

The mixed-effects models demonstrated numerical stability. In the biochemical-pregnancy model, increasing the number of adaptive Gauss–Hermite quadrature points from 15 to 30 produced identical regression coefficients, standard errors, random-intercept variance, and log-likelihood. The clinical-pregnancy estimates were also virtually unchanged. In the GEE sensitivity analysis for biochemical pregnancy, the 19–21 mm group remained associated with higher odds than the 17–18.9 mm group (OR 2.31, 95% CI 1.19–4.47; *p* = 0.013), whereas the >21.0 mm group was not significantly different than the reference group (OR 1.24, 95% CI 0.53–2.87; *p* = 0.617). The direction of the principal clinical-pregnancy findings was similarly preserved in the GEE analysis.

**Figure 2:**
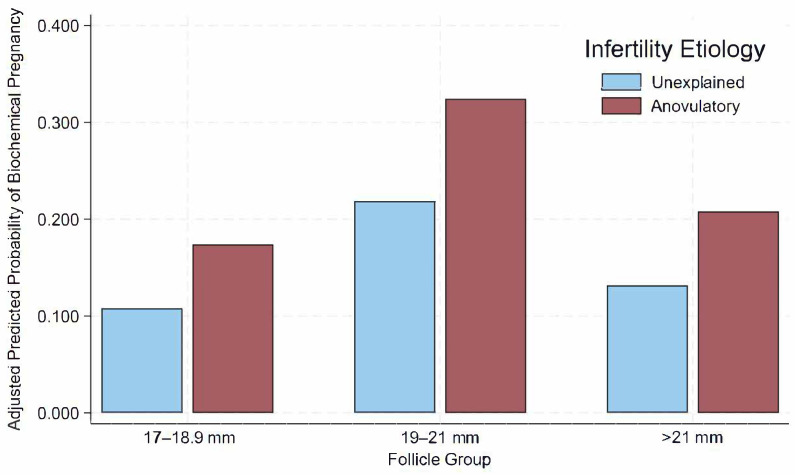
Adjusted predicted probabilities of biochemical pregnancy stratified by infertility etiology and dominant follicle-size group.

**Figure 3:**
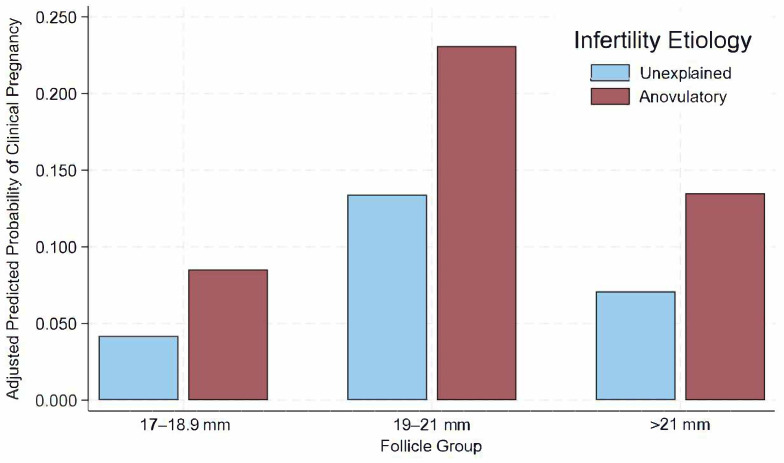
Adjusted predicted probabilities of clinical pregnancy stratified by infertility etiology and dominant follicle-size group.

**Figure 4:**
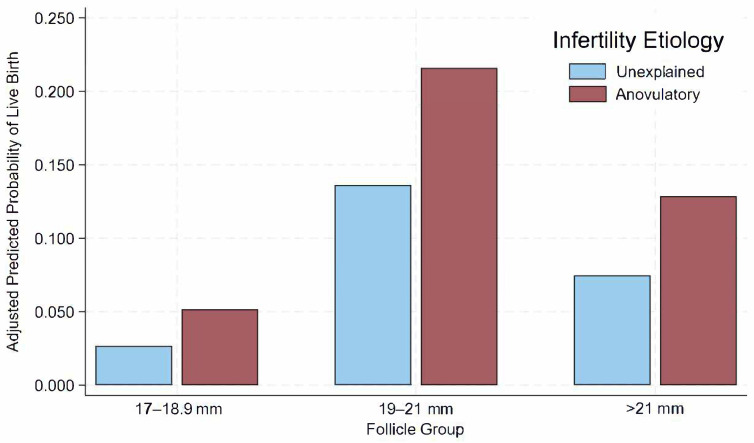
Adjusted predicted probabilities of live birth according to dominant follicle-size category and infertility etiology.

Adjusted predicted probabilities of biochemical pregnancy, clinical pregnancy, and live birth according to dominant follicle-size groups are summarized in Table 5. The adjusted predicted probabilities of biochemical pregnancy were 12.8% for the 17.0–18.9 mm group, 25.1% for the 19.0–21.0 mm group, and 15.5% for the >21.0 mm group. The corresponding probabilities were 5.5%, 16.4%, and 9.1% for clinical pregnancy and 3.5%, 16.1%, and 9.1% for live birth, respectively. After Bonferroni adjustment, pairwise comparisons demonstrated significant absolute differences between the 19.0–21.0 mm and 17.0–18.9 mm groups for biochemical pregnancy (absolute difference, 12.2 percentage points; *p* = 0.010), clinical pregnancy (10.8 percentage points; *p* = 0.001), and live birth (12.6 percentage points; *p* < 0.001). Comparisons between the >21.0 mm and 17.0–18.9 mm groups were not statistically significant for any outcome. Similarly, the differences between the >21.0 mm and 19.0–21.0 mm groups did not remain statistically significant after Bonferroni adjustment for biochemical pregnancy (*p* = 0.081), clinical pregnancy (*p* = 0.125), or live birth (*p* = 0.162). Adjusted predicted probabilities of biochemical, clinical pregnancy, and live birth stratified by infertility etiology and dominant follicle-size groups are illustrated in Figure 2, Figure 3 and Figure 4, respectively.

Sensitivity analyses were performed by restricting the dataset to participants who contributed a single IUI cycle. Participants with more than one IUI cycle were entirely excluded rather than selecting one cycle per participant. After excluding participants with repeated cycles, 334 participants, each contributing one cycle, remained in the restricted cohort. Biochemical pregnancy occurred in 89 cycles, clinical pregnancy in 55 cycles, and live birth in 55 cycles. The corresponding sensitivity analyses are presented in Supplementary Table S1 for biochemical pregnancy, Supplementary Table S2 for clinical pregnancy, and Supplementary Table S3 for live birth. The direction and magnitude of the associations between infertility type, follicle-size category, and reproductive outcomes were generally consistent with those observed in the primary mixed-effects analyses. Firth’s penalized logistic regression was used for the multivariable clinical-pregnancy and live-birth analyses to reduce potential small-sample bias. In the live-birth model, anovulatory infertility remained independently associated with higher odds of live birth (aOR 2.33, 95% CI 1.19–4.56; *p* = 0.013); the 19.0–21.0 mm group had higher odds of live birth than the 17.0–18.9 mm group (aOR 3.85, 95% CI 1.22–12.13; *p* = 0.021). Maternal age showed a borderline inverse association with live birth after adjustment (aOR 0.93, 95% CI 0.87–1.00; *p* = 0.060).

In sensitivity analyses modeling dominant follicle diameter as a continuous predictor, no significant linear association was observed with biochemical pregnancy (aOR per 1-mm increase, 1.01; 95% CI, 0.88–1.16; *p* = 0.858), clinical pregnancy (aOR, 1.01; 95% CI, 0.81–1.25; *p* = 0.933), or live birth (aOR, 1.07; 95% CI, 0.83–1.38; *p* = 0.601). The quadratic follicle-diameter term was also not statistically significant for biochemical pregnancy (β = −0.031; 95% CI, −0.088 to 0.025; *p* = 0.279), clinical pregnancy (β = −0.032; 95% CI, −0.121 to 0.057; *p* = 0.476), or live birth (β = −0.077; 95% CI, −0.194 to 0.039; *p* = 0.195). Furthermore, likelihood-ratio tests showed that inclusion of the quadratic term did not significantly improve model fit compared with the corresponding linear models (*p* = 0.262, *p* = 0.464, and *p* = 0.158, respectively). Thus, no significant linear or quadratic dose–response association between continuous follicle diameter and the reproductive outcomes was identified (Supplementary Table S4).

## 4. Discussion

In this retrospective cohort of 596 letrozole-induced ovulation inductions followed by IUI cycles, biochemical pregnancy, clinical pregnancy, and live birth were most frequent when the dominant follicle measured 19.0–21.0 mm at hCG triggering. Compared with 17.0–18.9 mm, this category was associated with higher adjusted odds of clinical pregnancy and live birth. The direction of the findings was preserved in population-averaged GEE and single-cycle sensitivity analyses. Nevertheless, the confidence intervals—particularly for live birth—were very wide and the continuous linear and quadratic follicle-diameter models were not statistically significant. The results, therefore, indicate a category-based association within this cohort rather than a validated biological threshold or a precise estimate of treatment benefit.

Biologically, dominant follicle diameter integrates granulosa-cell proliferation, follicular-fluid accumulation, steroidogenesis, cumulus expansion, and oocyte nuclear and cytoplasmic maturation. Triggering at a smaller diameter may precede full oocyte competence, whereas excessive delay may permit post-maturity or atresia and may disrupt synchronization between ovulation and endometrial receptivity. Letrozole’s short half-life and a lack of prolonged estrogen-receptor depletion may preserve endometrial development; however, trigger-day estradiol is often lower than in gonadotropin-stimulated cycles. The measured diameter may, therefore, not map identically to biological maturity across protocols. This provides a plausible explanation for a mid-range pattern; however, the present data cannot establish the mechanism.

The findings are broadly consistent with letrozole-specific studies by Ling et al., who reported a 19.1–21.0 mm range associated with clinical pregnancy and live birth, and by Cameron et al., who observed higher clinical pregnancy rates around 19–23 mm [13,16]. Liang et al. also reported different response patterns in ovulatory dysfunction and unexplained infertility [18]. Conversely, Chen et al. identified a smaller range in letrozole–HMG cycles, whereas Palatnik et al. reported a larger range in mixed clomiphene/letrozole protocols and highlighted an interaction with endometrial thickness [15,17]. Differences in stimulation agents, gonadotropin supplementation, spontaneous LH surge, trigger criteria, ultrasound timing, endometrial development, and infertility etiology probably contribute to the divergent cutoffs. The literature therefore supports protocol-specific interpretation rather than a universal follicle-size target.

The categorical analysis directly addressed clinically recognizable trigger ranges; however, categorization can create apparent boundaries and lose information. The absence of significant linear or quadratic associations in Supplementary Table S4 weakens any claim of a sharply defined optimum. Adjusted outcome probabilities were generally higher in anovulatory than unexplained infertility (Figure 2, Figure 3 and Figure 4); however, the full live-birth interaction model was not estimable because no live births occurred among 60 unexplained-infertility cycles in the 17.0–18.9 mm group. The restricted interaction estimate was nonsignificant and imprecise; therefore, this study does not establish etiology-specific trigger thresholds.

The strengths of this study include the relatively large cohort, a standardized letrozole-only protocol, use of patient-level random-intercept models for repeated cycles, Bonferroni-adjusted marginal pairwise comparisons, complete follow-up to live birth, and concordant GEE and single-cycle sensitivity analyses. Events-per-coefficient ratios were 18.4, 13.0, and 12.3 for biochemical pregnancy, clinical pregnancy, and live birth, respectively. Convergence, calibration, multicollinearity, and influential-cluster assessments were satisfactory. These internal diagnostics support numerical stability but do not eliminate overfitting or imprecision arising from sparse events, particularly in the smallest follicle-size group.

Several limitations remain. The retrospective, single-center design is susceptible to selection bias and residual confounding and limits generalizability to centers using other letrozole doses, gonadotropin supplementation, monitoring schedules, or trigger strategies. Smoking status, metabolic and endocrine comorbidities, previous fertility treatment, ovarian-reserve measures beyond AMH, semen morphology, sperm DNA fragmentation, and trigger-day estradiol, progesterone, and LH were unavailable or not consistently incorporated. Ultrasound measurements were operator-dependent. Embryo quality was not applicable because this study evaluated IUI rather than embryo transfer. Although live birth was included as a clinically meaningful secondary outcome with complete follow-up, it was not the prespecified primary outcome, and only three live births occurred in the 17.0–18.9 mm group, producing wide confidence intervals. Prospective multicenter studies with a prespecified live-birth endpoint, standardized hormonal and ultrasound monitoring, and etiology-stratified analyses are required.

## 5. Conclusions

In this retrospective single-center cohort, a dominant follicle size of 19.0–21.0 mm at hCG trigger was associated with higher biochemical pregnancy, clinical pregnancy, and live-birth rates than the 17.0–18.9 mm category. However, the live-birth estimate was imprecise, the continuous linear and quadratic analyses were not significant, and the observational design precludes causal inference. These findings are hypothesis-generating and do not establish an optimal trigger threshold. Prospective multicenter studies with prespecified live-birth endpoints and etiology-stratified analyses are needed before the results can be translated into clinical recommendations.

## Figures and Tables

**Table 1:** Baseline demographic, hormonal, and cycle characteristics according to dominant follicle-size groups.

Variables	Group I (*n* = 87 Cycles)	Group II (*n* = 416 Cycles)	Group III (*n* = 93 Cycles)	*p*-Value
Cycle Characteristics				
Age (years)	27.8 ± 3.8	28.4 ± 4.6	28.3 ± 4.9	0.942
BMI (kg/m^2^)	25.3 ± 3.7	26.2 ± 4.3	25.5 ± 4.0	0.262
Infertility duration (years)	2.0 (1.5–3.0)	2.0 (1.5–3.0)	2.0 (1.5–3.0)	0.362
Estradiol (pg/mL)	35.0 (28.0–45.0)	36.0 (28.5–45.0)	35.0 (26.0–44.0)	0.481
FSH (mIU/mL)	6.04 ± 1.35	6.14 ± 1.57	6.23 ± 1.61	0.124
LH (mIU/mL)	7.60 (6.00–9.90)	6.50 (5.20–9.00)	6.90 (5.05–9.00)	0.089
Prolactin (ng/mL)	18.0 (13.0–25.0)	18.0 (13.0–23.0)	17.0 (12.0–23.0)	0.135
TSH (mIU/L)	1.90 (1.28–2.50)	1.90 (1.50–2.80)	1.99 (1.50–2.70)	0.396
AMH (ng/mL)	3.70 (2.40–5.90)	3.42 (2.09–5.62)	3.05 (2.05–5.00)	0.021
Infertility etiology				0.686
Unexplained	60 (69.0%)	292 (70.2%)	69 (74.2%)	
Anovulatory	27 (31.0%)	124 (29.8%)	24 (25.8%)	
Subdominant follicular existence	51 (58.6%)	232 (55.8%)	48 (51.6%)	0.580
Ovulation characteristics				0.051
Mono-ovulation	58 (66.7%)	209 (50.2%)	50 (53.8%)	
Double ovulation	29 (33.3%)	207 (49.8%)	43 (46.2%)	
Endometrial thickness (mm)	8.21 ± 1.90	8.63 ± 2.09	8.55 ± 2.07	0.235
Ovulation induction duration (days)	10.8 ± 2.6	11.1 ± 2.4	11.1 ± 2.7	0.427
TMSC (×10^6^)	28.00 (9.03–40.00)	27.00 (11.00–49.00)	25.00 (10.00–44.00)	0.349
Biochemical pregnancy	11 (12.6%)	104 (25.0%)	14 (15.1%)	0.015
Clinical pregnancy	5 (5.8%)	65 (15.6%)	8 (8.6%)	0.012
Live birth	3 (3.4%)	63 (15.1%)	8 (8.6%)	0.008

Note: *p* values were obtained from mixed-effects regression models with patient ID included as a random intercept to account for repeated cycles within the same patient. Abbreviations: BMI, body mass index; FSH, follicle-stimulating hormone; LH, luteinizing hormone; TSH, thyroid-stimulating hormone; AMH, anti-Müllerian hormone; TMSC, total motile sperm count.

**Table 2:** Univariable and multivariable mixed-effects logistic regression analyses for predictors of biochemical pregnancy.

Variable	Univariable			Multivariable		
	OR	95% CI	*p*-Value	aOR	95% CI	*p*-Value
TSH (mIU/L)	0.972	0.790–1.196	0.790	-	-	-
Infertility type (anovulatory vs. unexplained)	1.970	1.250–3.105	0.003	1.794	1.080–2.978	0.024
Ovulation induction duration (days)	1.035	0.952–1.126	0.415	-	-	-
AMH (ng/mL)	1.067	0.998–1.140	0.057	1.005	0.930–1.086	0.903
Endometrial thickness (mm)	1.079	0.973–1.197	0.149	1.061	0.956–1.178	0.267
LH (mIU/mL)	1.041	0.978–1.109	0.208	-	-	-
TMSC (×10^6^)	1.007	0.999–1.015	0.096	1.005	0.997–1.014	0.216
Follicular diameter				-	-	-
19–21 mm vs. 17–18.9 mm	2.441	1.178–5.058	0.016	2.433	1.163–5.090	0.018
>21 mm vs. 17–18.9 mm	1.255	0.512–3.072	0.619	1.266	0.510–3.145	0.611
>21 mm vs. 19–21 mm	0.514	0.267–0.989	0.046	0.520	0.269–1.007	0.052
Infertility duration (years)	0.940	0.829–1.065	0.331	-	-	-
Age (years)	0.950	0.904–0.998	0.041	0.955	0.907–1.006	0.082
BMI (kg/m^2^)	1.022	0.971–1.076	0.400	-	-	-
FSH (mIU/mL)	0.921	0.800–1.061	0.253	-	-	-
Estradiol (pg/mL)	1.002	0.982–1.024	0.819	-	-	-
Prolactin (ng/mL)	0.990	0.966–1.013	0.388	-	-	-
Ovulation characteristics (double- vs. mono-ovulation)	1.128	0.739–1.723	0.578	-	-	-

Note: Odds ratios (ORs) and adjusted odds ratios (aORs) with 95% confidence intervals (CIs) were estimated using mixed-effects logistic regression models with patient ID included as a random intercept to account for repeated cycles within the same participant. Variables with *p* < 0.20 in univariable analyses and/or established clinical relevance were included in the multivariable model. Bold values indicate statistical significance (*p* < 0.05). Abbreviations: AMH, anti-Müllerian hormone; BMI, body mass index; CI, confidence interval; FSH, follicle-stimulating hormone; LH, luteinizing hormone; OR, odds ratio; TSH, thyroid-stimulating hormone; TMSC, total motile sperm count.

**Table 3:** Univariable and multivariable mixed-effects logistic regression analyses for predictors of clinical pregnancy.

Variable	Univariable			Multivariable		
	OR	95% CI	*p*-Value	aOR	95% CI	*p*-Value
TSH (mIU/L)	0.887	0.629–1.251	0.494	-	-	-
Infertility type (anovulatory vs. unexplained)	2.916	1.340–6.346	0.007	2.724	1.165–6.368	0.021
Ovulation induction duration (days)	1.111	0.959–1.287	0.162	1.037	0.893–1.205	0.632
AMH (ng/mL)	1.084	0.968–1.214	0.162	0.984	0.860–1.126	0.815
Endometrial thickness (mm)	1.049	0.886–1.242	0.578	-	-	-
LH (mIU/mL)	1.039	0.935–1.155	0.478	-	-	-
TMSC (×10^6^)	1.000	0.986–1.015	0.974	-	-	-
Follicular diameter				-	-	
19–21 mm vs. 17–18.9 mm	5.190	1.214–22.189	0.026	5.584	1.308–23.843	0.020
>21 mm vs. 17–18.9 mm	1.960	0.386–9.953	0.417	2.077	0.410–10.513	0.377
>21 mm vs. 19–21 mm	0.378	0.117–1.220	0.104	0.372	0.117–1.187	0.095
Infertility duration (years)	0.901	0.730–1.111	0.330			
Age (years)	0.911	0.833–0.995	0.039	0.917	0.836–1.006	0.067
BMI (kg/m^2^)	1.032	0.947–1.124	0.471	-	-	-
FSH (mIU/mL)	0.948	0.749–1.201	0.659	-	-	-
Estradiol (pg/mL)	1.015	0.980–1.050	0.408	-	-	-
Prolactin (ng/mL)	0.988	0.949–1.028	0.537	-	-	-
Ovulation characteristics (double- vs. mono-ovulation)	1.501	0.696–3.236	0.300	-	-	-

Note: Odds ratios (ORs) and adjusted odds ratios (aORs) with 95% confidence intervals (CIs) were estimated using mixed-effects logistic regression models with patient ID included as a random intercept to account for repeated cycles within the same participant. Variables with *p* < 0.20 in univariable analyses and/or established clinical relevance were included in the multivariable model. Bold values indicate statistical significance (*p* < 0.05). Abbreviations: AMH, anti-Müllerian hormone; BMI, body mass index; CI, confidence interval; FSH, follicle-stimulating hormone; LH, luteinizing hormone; OR, odds ratio; TSH, thyroid-stimulating hormone; TMSC, total motile sperm count.

**Table 4:** Univariable and multivariable mixed-effects logistic regression analyses of factors associated with live birth.

Variable	Univariable			Multivariable		
	OR	95% CI	*p*-Value	aOR	95% CI	*p*-Value
TSH (mIU/L)	1.224	0.485–3.089	0.669	-	-	-
Infertility type (anovulatory vs. unexplained)	2.636	1.289–6.690	0.010	2.711	1.002–7.336	0.050
Ovulation induction duration (days)	1.157	0.959–1.400	0.128	1.069	0.891–1.283	0.472
AMH (ng/mL)	1.113	0.966–1.283	0.138	1.004	0.853–1.182	0.961
Endometrial thickness (mm)	1.103	0.897–1.358	0.358	-	-	-
LH (mIU/mL)	1.057	0.925–1.207	0.416	-	-	-
TMSC (×10^6^)	1.004	0.987–1.021	0.667	-	-	-
Follicular diameter (continuous)	1.063	0.810–1.397	0.658			
Follicular diameter (categorical)				-	-	0.033
19–21 mm vs. 17–18.9 mm	14.100	1.507–131.892	0.020	15.149	1.739–131.950	0.014
>21 mm vs. 17–18.9 mm	4.575	0.470–44.554	0.190	4.987	0.548–45.418	0.154
>21 mm vs. 19–21 mm	0.324	0.075–1.407	0.133	0.329	0.082–1.325	0.118
Infertility duration (years)	0.893	0.695–1.146	0.373	-	-	-
Age (years)	0.887	0.791–0.996	0.042	0.898	0.800–1.009	0.070
BMI (kg/m^2^)	1.040	0.936–1.155	0.463	-	-	-
FSH (mIU/mL)	0.970	0.725–1.297	0.837	-	-	-
Estradiol (pg/mL)	1.024	0.978–1.072	0.319	-	-	-
Prolactin (ng/mL)	0.983	0.935–1.034	0.511	-	-	-
Ovulation characteristics (double- vs. mono-ovulation)	1.654	0.634–4.318	0.304	-	-	-

Note: Odds ratios (ORs) and adjusted odds ratios (aORs) with 95% confidence intervals (CIs) were estimated using mixed-effects logistic regression models with patient ID included as a random intercept to account for repeated cycles within the same participant. Variables with *p* < 0.20 in univariable analyses and/or established clinical relevance were included in the multivariable model. Bold values indicate statistical significance (*p* < 0.05). Abbreviations: AMH, anti-Müllerian hormone; BMI, body mass index; CI, confidence interval; FSH, follicle-stimulating hormone; LH, luteinizing hormone; OR, odds ratio; TSH, thyroid-stimulating hormone; TMSC, total motile sperm count.

**Table 5:** Adjusted predicted probabilities of biochemical pregnancy, clinical pregnancy, and live birth according to dominant follicle-size groups and pairwise comparisons.

Adjusted Predicted Probabilities (%)						
Follicle-Size Group	Biochemical Pregnancy		Clinical Pregnancy		Live Birth	
17–18.9 mm	12.8		5.5		3.5	
19–21 mm	25.1		16.4		16.1	
>21 mm	15.5		9.1		9.1	
Pairwise Comparisons						
Comparison	Biochemical Pregnancy		Clinical Pregnancy		Live Birth	
	Absolute Difference (%)	*p*-value *	Absolute Difference (%)	*p*-value *	Absolute Difference (%)	*p*-value *
19–21 mm vs. 17–18.9 mm	+12.2	0.010	+10.8	0.001	+12.6	<0.001
>21 mm vs. 17–18.9 mm	+2.7	>0.99	+3.5	>0.99	+5.7	0.359
>21 mm vs. 19–21 mm	−9.5	0.081	−7.3	0.125	−6.9	0.162

Note: Adjusted predicted probabilities and pairwise absolute differences were derived from the corresponding multivariable mixed-effects logistic regression models. * The *p* values for the three pairwise marginal-probability comparisons within each outcome were adjusted using the Bonferroni method.

## Data Availability

The data that support the findings of this study are available on request from the corresponding author. The data are not publicly available due to privacy or ethical restrictions.

## Supplementary Materials

The following supporting information can be downloaded at: https://www.mdpi.com/article/10.3390/jcm15155857/s1, Table S1, Univariable and multivariable logistic regression analyses for predictors of biochemical pregnancy among single-cycle participants; Table S2, Univariable standard and multivariable Firth penalized logistic regression analyses for predictors of clinical pregnancy among single-cycle participants; Table S3, Univariable standard and multivariable Firth penalized logistic regression analyses for predictors of live birth among single-cycle participants; Table S4, Sensitivity analyses evaluating dominant follicle diameter as a continuous linear and quadratic predictor of reproductive outcomes.

## Abbreviations

The following abbreviations are used in this manuscript:

AMH	anti-Müllerian hormone
aOR	adjusted odds ratio
BMI	body mass index
CC	clomiphene citrate
CI	confidence interval
FSH	follicle-stimulating hormone
hCG	human chorionic gonadotropin
HMG	human menopausal gonadotropin
IUI	intrauterine insemination
LH	luteinizing hormone
OI	ovulation induction
OR	odds ratio
PCOS	polycystic ovary syndrome
ROC	receiver-operating characteristic
TMSC	total motile sperm count
TSH	thyroid-stimulating hormone
TVUS	transvaginal ultrasonography

## References

[1] Zegers-Hochschild F., Adamson G.D., Dyer S., Racowsky C., de Mouzon J., Sokol R., Rienzi L., Sunde A., Schmidt L., Cooke I.D. (2017). The International Glossary on Infertility and Fertility Care, 2017. Fertil. Steril..

[2] Cox C.M., Thoma M.E., Tchangalova N., Mburu G., Bornstein M.J., Johnson C.L., Kiarie J. (2022). Infertility prevalence and the methods of estimation from 1990 to 2021: A systematic review and meta-analysis. Hum. Reprod. Open.

[3] Teede H.J., Tay C.T., Laven J.J.E., Dokras A., Moran L.J., Piltonen T.T., Costello M.F., Boivin J., Redman L.M., A Boyle J. (2023). Recommendations from the 2023 international evidence-based guideline for the assessment and management of polycystic ovary syndrome. Eur. J. Endocrinol..

[4] Committee on Gynecologic Practice, American College of Obstetricians and Gynecologists (2019). Infertility Workup for the Women’s Health Specialist. Obstet. Gynecol..

[5] Practice Committee of the American Society for Reproductive Medicine (2020). Evidence-based treatments for couples with unexplained infertility: A guideline. Fertil. Steril..

[6] Cole P.A., Robinson C.H. (1990). Mechanism and inhibition of cytochrome P-450 aromatase. J. Med. Chem..

[7] Legro R.S., Brzyski R.G., Diamond M.P., Coutifaris C., Schlaff W.D., Casson P., Christman G.M., Huang H., Yan Q., Alvero R. (2014). Letrozole versus Clomiphene for Infertility in the Polycystic Ovary Syndrome. N. Engl. J. Med..

[8] Miller K.A.R., Monteiro G. (2025). Comparative Outcomes of Letrozol Versus Clomiphene Citrate for Ovulation Induction in Patients with Pcos: A Systematic Review and Meta-Analysis. Fertil. Steril..

[9] Abu-Zaid A., Gari A., Sabban H., Alshahrani M.S., Khadawardi K., Badghish E., AlSghan R., Bukhari I.A., Alyousef A., Abuzaid M. (2024). Comparison of Letrozole and Clomiphene Citrate in Pregnancy Outcomes in Patients with Polycystic Ovary Syndrome: A Systematic Review and Meta-analysis. Reprod. Sci..

[10] Huniadi A., Bimbo-Szuhai E., Botea M., Zaha I., Beiusanu C., Pallag A., Stefan L., Bodog A., Șandor M., Grierosu C. (2023). Fertility Predictors in Intrauterine Insemination (IUI). J. Pers. Med..

[11] Hansen K.R., He A.L.W., Styer A.K., Wild R.A., Butts S., Engmann L., Diamond M.P., Legro R.S., Coutifaris C., Alvero R. (2016). Predictors of pregnancy and live-birth in couples with unexplained infertility after ovarian stimulation–intrauterine insemination. Fertil. Steril..

[12] Souter I., Sun F., Zhang H., Diamond M.P., Legro R.S., Wild R.A., Hansen K.R., Santoro N. (2022). A personalized medicine approach to ovulation induction/ovarian stimulation: Development of a predictive model and online calculator from level-I evidence. Fertil. Steril..

[13] Ling L., Xia D., Jin Y., Hong R., Wang J., Liang Y. (2024). Effect of follicle size on pregnancy outcomes in patients undergoing first letrozole-intrauterine insemination. Eur. J. Med. Res..

[14] Guo Z., Chen S., Chen Z., Hu P., Hao Y., Yu Q. (2023). Predictors of response to ovulation induction using letrozole in women with polycystic ovary syndrome. BMC Endocr. Disord..

[15] Chen L., Jiang S., Xi Q., Li W., Lyu Q., Kuang Y. (2023). Optimal lead follicle size in letrozole human menopausal gonadotrophin intrauterine insemination cycles with and without spontaneous LH surge. Reprod. Biomed. Online.

[16] Cameron K., Abu-Rafea B., Vilos A., Hollett-Caines J., Rebel M., Van Oirschot M., Mourad A. (2025). Optimal follicle size for human chorionic gonadotropin trigger in mono-follicular growth during letrozole-induced intrauterine insemination cycles: Findings from a large retrospective study. Fertil. Steril..

[17] Palatnik A., Strawn E., Szabo A., Robb P. (2012). What is the optimal follicular size before triggering ovulation in intrauterine insemination cycles with clomiphene citrate or letrozole? An analysis of 988 cycles. Fertil. Steril..

[18] Liang Y., Lin H., Qiu Q., Pan P., Jiao X., Li Y., Zhang Q. (2025). Differential optimal follicle sizes for ovulatory dysfunction and unexplained infertility in LE-IUI cycles: A retrospective analysis. Eur. J. Med. Res..

