# Supplementary material for: Dominant Follicle Size as a Predictor of Pregnancy in Letrozole Intrauterine Insemination
Source: J Clin Med. 2026 Jul 27;15(15):5857. doi: 10.3390/jcm15155857 (PMC13466813; doi:10.3390/jcm15155857)
Supplement: Supplementary file 1 [file jcm-15-05857-s001.zip › Table S1.pdf]

Supplementary Table S1. Univariable and multivariable binary logistic regression analyses for predictors of biochemical pregnancy in single-cycle participants

Variable	Univariable			Multivariable		
	OR	95% CI	p-value	aOR	95% CI	p-value
TSH (mIU/L)	0.865	0.666-1.123	0.275	-	-	-
Infertility type (anovulatory vs unexplained)	2.424	1.465-4.010	0.001	2.202	1.224-3.960	0.008
Ovulation induction duration (days)	1.038	0.950-1.134	0.412	-	-	-
AMH (ng/mL)	1.079	1.002-1.161	0.043	1.008	0.916-1.110	0.863
Endometrial Thickness (mm)	1.155	1.022-1.306	0.021	1.151	1.012-1.309	0.033
LH (mIU/mL)	1.058	0.990-1.131	0.094	-	-	-
TMSC ($\times 10^6$)	1.002	0.993-1.011	0.647	-	-	-
Follicular diameter						
19–21 mm vs 17–18.9 mm	3.463	1.407-8.523	0.007	3.943	1.651-9.416	0.002
>21mm vs 17–18.9 mm	1.487	0.508-4.356	0.469	1.518	0.509-4.527	0.454
>21mm vs 19–21 mm	0.430	0.211-0.873	0.019	0.385	0.176-0.843	0.017
Infertility Duration (years)	0.900	0.762-1.063	0.215	0.966	0.807-1.157	0.707
Age (years)	0.941	0.891-0.994	0.029	0.942	0.885-1.003	0.062
BMI (kg/m^2)	1.026	0.963-1.093	0.431	-	-	-
FSH (mIU/mL)	0.913	0.773-1.079	0.285	-	-	-
Estradiol (pg/mL)	0.997	0.974-1.020	0.777	-	-	-
Prolactin (ng/mL)	1.004	0.981-1.028	0.722	-	-	-
Ovulation Characteristics (Double vs mono ovulation)	1.282	0.788-2.087	0.317	-	-	-

Note: Odds ratios (ORs) and adjusted odds ratios (aORs) with 95% confidence intervals (CIs) were estimated using standard binary logistic regression models. This sensitivity analysis included only participants who contributed a single IUI cycle; participants with repeated cycles were excluded to eliminate within-subject correlation. Variables with $p < 0.20$ in univariable analyses and/or established clinical relevance were included in the multivariable model. Bold values indicate statistical significance ($p < 0.05$).

Abbreviations: AMH, anti-Müllerian hormone; BMI, body mass index; CI, confidence interval; FSH, follicle-stimulating hormone; LH, luteinizing hormone; OR, odds ratio; aOR, adjusted odds ratio; PRL, prolactin; TMSC, total motile sperm count; TSH, thyroid-stimulating hormone.
