# Supplementary material for: Dominant Follicle Size as a Predictor of Pregnancy in Letrozole Intrauterine Insemination
Source: J Clin Med. 2026 Jul 27;15(15):5857. doi: 10.3390/jcm15155857 (PMC13466813; doi:10.3390/jcm15155857)
Supplement: Supplementary file 1 [file jcm-15-05857-s001.zip › Table S2.pdf]

Supplementary Table S2. Univariable and multivariable (Firth's penalized) binary logistic regression analyses for predictors of clinical pregnancy in single-cycle participants

Variable	Univariable			Multivariable†		
	OR	95% CI	p-value	aOR	95% CI	p-value
TSH (mIU/L)	0.821	0.593-1.136	0.235	0.796	0.589-1.075	0.136
Infertility type (anovulatory vs unexplained)	2.580	1.430-4.653	0.002	2.374	1.208-4.667	0.012
Ovulation induction duration (days)	1.051	0.949-1.165	0.341	-	-	-
AMH (ng/mL)	1.070	0.989-1.158	0.092	0.988	0.889-1.097	0.816
Endometrial Thickness (mm)	1.117	0.973-1.282	0.116	1.105	0.960-1.273	0.165
LH (mIU/mL)	1.059	0.978-1.148	0.158	-	-	-
TMSC ($x10^6$)	0.999	0.988-1.010	0.847	-	-	-
Follicular diameter						
19–21 mm vs 17–18.9 mm	3.920	1.164-13.210	0.028	3.999	1.263-12.660	0.018
>21mm vs 17–18.9 mm	1.883	0.460-7.707	0.379	1.830	0.475-7.048	0.380
>21mm vs 19–21 mm	0.480	0.205-1.126	0.092	0.458	0.194-1.078	0.074
Infertility Duration (years)	0.847	0.691-1.038	0.109	0.919	0.740-1.139	0.437
Age (years)	0.921	0.857-0.991	0.027	0.929	0.864-0.997	0.042
BMI (kg/m^2)	1.029	0.955-1.110	0.450	-	-	-
FSH (mIU/mL)	0.948	0.786-1.144	0.579	-	-	-
Estradiol (pg/mL)	1.005	0.979-1.033	0.687	-	-	-
Prolactin (ng/mL)	1.008	0.982-1.035	0.559	-	-	-
Ovulation Characteristics (Double vs mono ovulation)	1.335	0.747-2.384	0.329	-	-	-

† Multivariable estimates obtained using Firth's penalized likelihood.

Note: Odds ratios (ORs) and adjusted odds ratios (aORs) with 95% confidence intervals (CIs) were estimated using standard binary logistic regression for univariable analyses and Firth's penalized logistic regression for multivariable analyses to reduce small-sample bias and address low events-per-variable ratios. This sensitivity analysis included only participants who contributed a single IUI cycle; participants with repeated cycles were excluded. Variables with $p < 0.20$ in univariable analyses and/or established clinical relevance were included in the multivariable model. Bold values indicate statistical significance ($p < 0.05$).

Abbreviations: AMH, anti-Müllerian hormone; BMI, body mass index; CI, confidence interval; FSH, follicle-stimulating hormone; LH, luteinizing hormone; OR, odds ratio; aOR, adjusted odds ratio; PRL, prolactin; TMSC, total motile sperm count; TSH, thyroid-stimulating hormone.
