# Supplementary material for: Dominant Follicle Size as a Predictor of Pregnancy in Letrozole Intrauterine Insemination
Source: J Clin Med. 2026 Jul 27;15(15):5857. doi: 10.3390/jcm15155857 (PMC13466813; doi:10.3390/jcm15155857)
Supplement: Supplementary file 1 [file jcm-15-05857-s001.zip › Tables S3 and S4.pdf]

Supplementary Table S3. Univariable standard and multivariable Firth penalized logistic regression analyses for predictors of live birth among single-cycle participants

Variable	Univariable			Multivariable		
	OR	95% CI	p-value	aOR	95% CI	p-value
TSH (mIU/L)	0.82	0.61–1.11	0.202	-	-	-
Infertility type (anovulatory vs unexplained)	2.58	1.43–4.65	0.002	2.33	1.19–4.56	0.013
Ovulation induction duration (days)	1.05	0.94–1.17	0.361	-	-	-
AMH (ng/mL)	1.07	0.99–1.16	0.105	0.98	0.88–1.09	0.700
Endometrial Thickness (mm)	1.12	0.97–1.29	0.122	1.10	0.96–1.27	0.174
LH (mIU/mL)	1.06	0.98–1.15	0.148	1.04	0.96–1.14	0.340
TMSC (×10^6)	1.00	0.99–1.01	0.858	-	-	-
Follicular diameter						
19–21 mm vs 17–18.9 mm	3.92	1.17–13.19	0.027	3.85	1.22–12.13	0.021
>21mm vs 17–18.9 mm	1.88	0.46–7.69	0.378	1.78	0.46–6.83	0.401
>21mm vs 19–21 mm	0.48	0.21–1.12	0.091	0.46	0.20–1.09	0.077
Infertility Duration (years)	0.85	0.69–1.04	0.109	0.92	0.74–1.14	0.450
Age (years)	0.92	0.86–0.99	0.017	0.93	0.87–1.00	0.060
BMI (kg/m²)	1.03	0.96–1.10	0.412	-	-	-
FSH (mIU/mL)	0.95	0.78–1.15	0.591	-	-	-
Estradiol (pg/mL)	1.01	0.98–1.03	0.709	-	-	-
Prolactin (ng/mL)	1.01	0.98–1.04	0.594	-	-	-
Ovulation Characteristics (Double vs mono ovulation)	1.33	0.75–2.38	0.329	-	-	-

Note: Univariable odds ratios (ORs) were estimated using standard binary logistic regression, whereas adjusted odds ratios (aORs) were estimated using Firth's penalized logistic regression. This sensitivity analysis included only participants who contributed a single IUI cycle; participants with repeated cycles were excluded to eliminate within-subject correlation. The multivariable model included infertility type, AMH level, endometrial thickness, LH level, follicle-size category, infertility duration, and maternal age. Dashes indicate variables not included in the multivariable model. Bold values indicate statistical significance ($p < 0.05$).

Abbreviations: AMH, anti-Müllerian hormone; BMI, body mass index; CI, confidence interval; FSH, follicle-stimulating hormone; LH, luteinizing hormone; OR, odds ratio; aOR, adjusted odds ratio; PRL, prolactin; TMSC, total motile sperm count; TSH, thyroid-stimulating hormone.

Supplementary Table S4. Sensitivity analyses evaluating dominant follicle diameter as a continuous linear and quadratic predictor of reproductive outcomes

Outcome	Linear model		Quadratic model		Likelihood-ratio model comparison	
	aOR per 1-mm increase (95% CI)	p-value	Quadratic term, β (95% CI)	p-value	LR $\chi^2(1)$	p-value
Biochemical pregnancy	1.01 (0.88–1.16)	0.858	−0.031 (−0.088 to 0.025)	0.279	1.26	0.262
Clinical pregnancy	1.01 (0.81–1.25)	0.933	−0.032 (−0.121 to 0.057)	0.476	0.54	0.464
Live birth	1.07 (0.83–1.38)	0.601	−0.077 (−0.194 to 0.039)	0.195	2.00	0.158

Note: Estimates were obtained from mixed-effects logistic regression models with patient identifier included as a random intercept and 30 adaptive Gauss–Hermite integration points. Linear models report adjusted odds ratios per 1-mm increase in dominant follicle diameter. Quadratic-term coefficients are presented on the log-odds scale. The likelihood-ratio test compared each quadratic model with the corresponding nested linear model. Biochemical pregnancy models were adjusted for infertility etiology, anti-Müllerian hormone level, endometrial thickness, total motile sperm count, and maternal age. Clinical pregnancy and live-birth models were adjusted for infertility etiology, ovulation-induction duration, anti-Müllerian hormone level, and maternal age.

Abbreviations: aOR, adjusted odds ratio; CI, confidence interval; LR, likelihood-ratio.
